# Music in Quarantine: Connections Between Changes in Lifestyle, Psychological States, and Musical Behaviors During COVID-19 Pandemic

**DOI:** 10.3389/fpsyg.2021.689505

**Published:** 2021-10-11

**Authors:** Hiroko Terasawa, Masaki Matsubara, Visda Goudarzi, Makiko Sadakata

**Affiliations:** ^1^Faculty of Library, Information and Media Science, University of Tsukuba, Tsukuba, Japan; ^2^Audio Arts and Acoustics Department, Columbia College Chicago, Chicago, IL, United States; ^3^Institute for Logic, Language and Computation, University of Amsterdam, Amsterdam, Netherlands

**Keywords:** COVID-19, music, stress, lifestyle, emotion, media usage, housing, musicianship

## Abstract

Music is not only the art of organized sound but also a compound of social interaction among people, built upon social and environmental foundations. Since the beginning of the COVID-19 outbreak, containment measures such as shelter-in-place, lockdown, social distancing, and self-quarantine have severely impacted the foundation of human society, resulting in a drastic change in our everyday experience. In this paper, the relationships between musical behavior, lifestyle, and psychological states during the shelter-in-place period of the COVID-19 pandemic are investigated. An online survey on musical experience, lifestyle changes, stress level, musical behaviors, media usage, and environmental sound perception was conducted. The survey was conducted in early June 2020. Responses from 620 people in 24 countries were collected, with the large proportion of the responses coming from the U.S. (55.5%) and India (21.4%). Structural equation modeling (SEM) analysis revealed causal relationships between lifestyle, stress, and music behaviors. Elements such as stress-level change, work risk, and staying home contribute to changes in musical experiences, such as moderating emotion with music, feeling emotional with music, and being more attentive to music. Stress-level change was correlated with work risk and income change, and people who started living with others due to the outbreak, especially with their children, indicated less change in stress level. People with more stress-level change tended to use music more purposefully for their mental well-being, such as to moderate emotions, to influence mood, and to relax. In addition, people with more stress-level change tend to be more annoyed by neighbors' noise. Housing type was not directly associated with annoyance; however, attention to environmental sounds decreased when the housing type was smaller. Attention to environmental and musical sounds and the emotional responses to them are highly inter-correlated. Multi-group SEM based on musicians showed that the causal relationship structure for professional musicians differs from that of less-experienced musicians. For professional musicians, staying at home was the only component that caused all musical behavior changes; stress did not cause musical behavior changes. Regarding Internet use, listening to music via YouTube and streaming was preferred over TV and radio, especially among less-experienced musicians, while participation in the online music community was preferred by more advanced musicians. This work suggests that social, environmental, and personal factors and limitations influence the changes in our musical behavior, perception of sonic experience, and emotional recognition, and that people actively accommodated the unusual pandemic situations using music and Internet technologies.

## 1. Introduction

### 1.1. The Pandemic and Shift in Our Musical Life

The COVID-19 pandemic has caused significant lifestyle changes around the world (Bavel et al., 2020; Gao and Scullin, 2020; Ammar et al., 2021): many have experienced financial difficulties due to unemployment or inactivity (ILO, 2020), many have had more intensive childcare responsibilities that before (Blum, 2020; Rocco, 2020), and many have suffered from loneliness, worry, or depression (Li and Wang, 2020; Salari et al., 2020). Social distancing and shelter-in-place (SiP) have entirely changed the dynamics and form of our social lives. While essential workers continued their commutes, many others were encouraged to work from home. In addition, schools were closed in many countries, and children stayed home with their families. Consequently, there has been less commuting and local travel (Elldér, 2020). In this paper, we aim to portray the shift in our musical life along with the lifestyle and social changes at the time of the early shelter-in-place (SiP) period of the COVID-19 pandemic.

Such changes have a significant impact on various aspects of our everyday life, including musical behaviors, that is, the activities related to music, that traditionally involve social interactions and gatherings among performers and listeners (Benzon, 2001; Tarr et al., 2014; Terasawa et al., 2019). SiP suppressed people's direct interactions for musical purposes, such as in-person rehearsals, in-person lessons, concerts (both performing and attending), and outdoor music festivals (Hall, 2020; Robinson, 2020). Children who usually attend daycare or school but stayed at home during SiP lost opportunities to sing, play, and dance with their peers (Daubney and Fautley, 2020; Sherwood, 2020).

Sloboda (2010) describe everyday music as something that is experienced in ordinary places, such as in the home, workplace, public transport, restaurants, shopping malls, etc., but not in concert halls. The paper also discuss that emotional experiences with everyday music are more frequent, less intense, and less memorable. Notably, even such non-eventful music occasions are lost in SiP, such as going out to pubs with live music, listening to music or radio during the commute, and encountering popular tunes in shops. As such, the shift in location and duration of everyday music experience could, in turn, cause changes in musical behaviors, such as the type of activities, duration, and emotional responses.

Among music activities, listening to music is most accessible for many people. The choices of devices and formats for music listening are expanding (Krause et al., 2015), offering more flexible styles and opportunities for doing so. We listen to music for many different purposes in our everyday life, including moderating mood and emotion (Thoma et al., 2012), boosting concentration (Shih et al., 2009; Huang and Shih, 2011), driving (Dibben and Williamson, 2007), and sleeping (Lai and Good, 2006; Harmat et al., 2008), to name a few. We also listen to music as a background for various daily tasks, such as running, exercising, housework, commuting, studying, and working (Lesiuk, 2005; Kämpfe et al., 2011). While there are large individual differences in the way people use music (Chamorro-Premuzic and Furnham, 2007), previous works have highlighted the common social functions of music usage, such as self-identity (self-awareness), interpersonal relationships (social connections), and mood regulation (Hargreaves and North, 2007; Schäfer et al., 2013). In particular, Saarikallio et al. provided an insightful breakdown of mood regulation strategies, such as entertainment, revival, strong sensation, diversion, discharge, mental work, and solace (Saarikallio and Erkkilä, 2007; Saarikallio, 2008, 2011). Notably, listening to music by oneself seemed to work in all of the above strategies. Thus, among music activities, listening to music is not only the most accessible, but also a versatile method to regulate mood. During the pandemic, many people face psychological difficulties, such as stress, anxiety, and depression (Li and Wang, 2020; Salari et al., 2020). These conditions inevitably influence individuals' basic mood as well as their self- and interpersonal relationships. The change in psychological and social circumstances is reflected in the way people use music in everyday life. For example, music listening time has typically increased during the pandemic (Cabedo-Mas et al., 2021; Carlson et al., 2021; Fink et al., 2021; Hurwitz and Krumhansl, 2021). The increase may be related to another common finding that people listen to music more frequently to cope with stress, regulate moods and emotions, and connect with others during the pandemic than under usual circumstances (Cabedo-Mas et al., 2021; Fink et al., 2021; Granot et al., 2021; Henry et al., 2021; Krause et al., 2021; Ribeiro et al., 2021b; Vidas et al., 2021; Ziv and Hollander-Shabtai, 2021). Remarkably, there are similar observations worldwide, highlighting that music not only serves such functions but is also one of the most common methods for this purpose during the pandemic.

Taken together, a forced change in lifestyle and situation should have a significant impact on people's musical life. Therefore, the primary aim of this study is to investigate the interrelation of musical behaviors, lifestyle, and psychological states during SiP.

### 1.2. Environmental Sounds

A portion of the questionnaire used in this study was dedicated to questions about environmental sounds and noise from the neighborhood. We asked about them because we regard environmental sounds and noises as a part and a foundation of musical experiences at home.

There has been a thread of important musical works and theories about the intersection of music and noise: The notable works in this direction include É*tude aux chemins de fer* by Pierre Schaeffer (1948), *Déserts* by Edgard Varèse (1950–1954), and *Presque rien No.1 - le lever du jour au bord de la mer* by Luc Ferrari (1967–1970). Composers such as Luigi Russolo, Pierre Schaeffer, Michel Chion, and Trevor Wishart not only used everyday noises as material for their music but also wrote their manifesto and theories in their books (Russolo, 1916; Schaeffer, 1966; Chion, 1983; Wishart, 1996). The degree of abstraction in treating noise and environmental sounds as music, that is, if the identity of a sound source has some role in the music, can differ for each artist and each work. Some composers emphasize the referential approach, in which the environmental sounds and noises are intended to bring some image about the sound source, or to refer to iconic soundscapes or musical signs (Norman, 1996; Truax, 1996). While these works represent the attitude of integrating noise and environmental sounds into music, another movement is to bring musical ears into the perception of environmental sounds. For example, Murray Schafer proposed the concept of “soundscape,” in which he claims that ordinary environmental sounds and noises are worth listening to with musical attention (Schafer, 1977). With this background, we view environmental sounds and noise as a part of musical culture.

Moreover, noise acoustics studies have shown that environmental sounds and noises interact with the listeners' psychological states (Kroesen et al., 2010; Schreckenberg et al., 2010; Hammersen et al., 2016; Jensen et al., 2019; Tao et al., 2020). During SiP, exposure to environmental sounds and noises around the house increased because of the longer time spent at home. As previously discussed, basic mood and musical emotions are considered to interact. From that perspective, considering environmental sounds and noises may help to better understand musical behaviors and perceptions during SiP.

### 1.3. Musicianship

Considering the interaction of changes in musical behaviors, lifestyle, and psychological states, we speculate that musicianship may play an important role. Many musicians spend a long time practicing music at a young age (Jørgensen, 2003), and music training seems to be accompanied by many transfer effects such as listening skills, fine motor skills, and temporal processing (Jakobson and Cuddy, 2019). Musicians are reported to have better speech in noise perception than non-musicians (Coffey et al., 2017), better auditory segregation of simultaneously occurring sounds (Zendel and Alain, 2009), and better recognition of environmental sounds (Lemaitre et al., 2010). Resnicow et al. (2004) suggested that everyday emotional intelligence and emotion-recognition skills in music are correlated. These studies suggest that the musicians' lifestyle, perception of music, and emotional experience can be quite different from those of non-musicians.

Another element we anticipate seeing is the effect of musicianship on media usage. In recent years, online music-streaming services have become quite popular and have affected record sales (Kretschmer and Peukert, 2020; Lee et al., 2020) and professional musicians' survival strategies (Kaimann et al., 2021). During the pandemic, this shift from live events to online services has been even more accelerated (Camilleri and Falzon, 2020; Coman, 2020; Sun et al., 2020; Boyce et al., 2021). These studies suggest that many people sought artistic and entertainment content on the Internet, and perhaps even more so during the pandemic.

While non-musicians enjoyed the musical content available online, professional musicians were running out opportunities for physical performances and struggling to maintain their activities. Many professional musicians have started online musical events, such as performance streaming via social media (UNRIC, 2020). Balcony performances, with musicians singing or playing from a balcony or a courtyard to share their music with community members have gone viral on the Internet (Calvo and Bejarano, 2020; Langley and Coutts, 2020). Also, many music teachers made a shift from conducting physical lessons to online lessons (de Bruin, 2021).

Musicians pursued the Internet's capacity not only as a place for final presentations but also for other processes such as rehearsals and lessons (Daffern and Brereton, 2021), planning and production (Fram et al., 2021), and improvisation (MacDonald et al., 2021), to name a few. Such proactive attitudes toward using the Internet for musical purposes are quite different from passively consuming musical content available online. Therefore, we expected people to display different balances between proactive and passive Internet usage for music, depending on their level of musicianship.

### 1.4. The Current Study

This paper explores how our musical and soundscape experience evolved during the first wave of the COVID-19 pandemic in early 2020, along with other changes in lifestyles and psychological states. In June 2020, during the very first wave of the pandemic, we distributed an international online questionnaire, and 620 people from 24 countries participated. The questionnaire asked about participants' basic backgrounds, COVID-19 situations, housing environments, lifestyle changes, work-style changes, media usage, and musical experiences, resulting in a rich and extensive dataset. Our main analyses focused on highlighting a potential causal relationship among key components of musical behaviors, stress, and lifestyle by using structural equation modeling (SEM) (Ullman and Bentler, 2012). SEM is a general multivariate statistical framework that considers the relationship between the observed and latent variables. In addition to the SEM analysis, detailed follow-up reports on the relationship between stress, the use and experience of music, and the effect of musicianship, as well as media usage and perception of environmental sounds during SiP are provided in the following sections.

## 2. Methods

### 2.1. Questionnaire

In this survey, participants were asked to provide demographic data and to complete 83 questions related to the COVID-19 situation, lifestyle changes, perception of environmental sounds, musical behavior changes, and media usage. All the questions are listed in Appendix 1 in Supplementary Material, along with the question numbers, variable names, and format of answers. In the following sections, each question is referred to by its number, such as Q1 for Question 1. Table 1 shows the key questions from the full list, which were identified as dominant factors in the factor analysis conducted in section 3.2. Most of the responses were collected using a 7-point Likert scale (LS-7; with the score values 1-much less, 4-no change, 7-much more) and a 4-point positive-sided ordinal scale (PS-4; with the score values 1-Not at all, 4-very much), and some questions were asked in the free description of checklist format. In the following sections, the mean, standard deviation (SD), and median were calculated based on the scale values of the given format. For the LS-7 and PS-4 questions, the mean, SD, and median are provided with the list of questions in Appendix 1.

**Table 1:** List of key questions: Question sentences and numbers, response formats, and variable names.

**Question**	**#**	**Format**	**Variable name**
Do you stay at home more or less than before, since the COVID-19 outbreak?	14	LS-7	StayHome
Are you a musician (e.g., playing musical instruments, singing, composing, DJ, audio-engineering, theorist, etc.)?	15	PS-4	Musician
Do you get distracted by the residents of your household?	25	PS-4	DistractionResidents
How clearly do you hear your neighbors' sounds in your residence?	28	PS-4	NeighborsNoiseLevel
Are you more annoyed with the environmental sound from neighbors (noise, footsteps, voices, pets, etc.) than before the COVID 19 outbreak?	29	LS-7	NeighborsSoundAnnoyance
How did your level of stress change after the outbreak?	30	LS-7	StressLevelChange (SLC)
Do you feel more risk with your work, such as layoffs, infections, and other factors, due to the outbreak?	31	LS-7	WorkRisk
During the SiP peak time (the most strict time of measures against COVID-19), did (do) you spend longer time listening to music than before the outbreak?	33	LS-7	LongerMusicListening
Did (do) you become more attentive to environmental sounds (e.g., traffic sounds, birds, noise from neighbors, noise in your residence, etc.) during SiP than before?	36	LS-7	AttentiveEnvSound
Did (do) you listen to music in your private time during the SiP peak time?	43	PS-4	MusicListeningPrivate
Did (do) you feel more emotional when you listen to music during the SiP peak time?	46	LS-7	EmotionalWithMusic
Did (do) you pay more attention when you listen to music during the SiP peak time?	47	LS-7	AttentiveMusic
During the SiP peak time, did (do) you use music to moderate emotions or change your mood more often than before?	50	LS-7	ModerateEmotionWithMusic
How much did (do) you play, sing, dance, play music-performing games (e.g., music-focused video games) during SiP?	51	LS-7	MusicalActivities

The questionnaire took approximately 20–30 min to complete. The study was approved by the Ethics Committee of the Faculty of Library, Information and Media Science, University of Tsukuba (No. 20-6). The survey was conducted in early June 2020.

### 2.2. Participants

The questionnaire was prepared using Google Forms. A total of 628 participants completed the survey. Participants were recruited through Amazon Mechanical Turk (AMT, USD 3.5, as a reward) and the researchers' community mailing lists and acquaintances (Auditory mailing list^1^, SMC-network^2^, ICAD mailing list^3^, Onsei-mail^4^, ASJ-onkyonet^5^, JSSA mailing list^6^, JSMPC mailing list ^7^, and their acquaintances, which we denote as the community). The e-mail invitation briefly stated the aim of the study^8^. Among the 628 complete responses, eight participants were excluded because they reported spending more than 168 hours a week (i.e., more than 24 hours a day) on either work, family-related, or leisure activities. After removing unreliable data, a total of 620 responses were analyzed. Among them, 470 responses (76%) were from AMT, and 150 responses (24%) were from the community. Some U.S. residents reported other countries as their country of residence, and they were manually corrected by referencing postal codes.

### 2.3. Statistical Methods

Factor analysis and SEM were used to quantitatively investigate the structure (i.e., potentially causal relationships) in response to multiple questions.

Factor analysis was used to identify latent variables and observed variables that significantly contributed to latent variables. Based on these, the core structure of a SEM model was constructed to describe the potential causal relationships among them. Finally, an exploratory SEM (Asparouhov and Muthén, 2009) was performed to find the best-fitting model by examining all the relations between the latent variables.

In the other analyses, non-parametric tests were used to compare groups; Mann-Whitney U test to compare two groups, Kruskal-Wallis ANOVA to compare more than two groups, followed the Dunn's test for multiple comparison with α <0.05, and Spearman's ρ to examine the correlations. Bonferroni correction was applied when the degree of freedom (df) or the number of comparison is smaller than 6, Holm-Bonferroni correction was applied when greater than 6.

## 3. Results

### 3.1. General Summary

Table 2 shows participants' gender, age group, and country. A large proportion (76%) of the participants were between the ages of 20 and 39. The responses of 620 people from 24 countries were analyzed in this study, and 55.5% of the participants were from the U.S., followed by 21.4% from India. Since the number of participants from each country varied greatly, we refrained from conducting country-wise analyses. Over 95% of our participants indicated that they liked music (moderately 20.8%, very much 76.4%). In addition, many of them also had musical experience, as shown in Table 3.

**Table 2:** Gender, age, and country distributions of the responses.

**Group**	**Number of participants**	**Rate (%)**
**Gender**		
Female	231	37.3
Male	386	62.2
Other	3	0.5
**Age (years old)**		
13–19	1	0.16
20–29	240	38.7
30–39	203	32.7
40–49	111	17.9
50–59	38	6.1
60–69	20	3.2
70–79	6	0.97
80–89	1	0.16
**Country**		
Brazil	8	1.3
Canada	7	1.1
Germany	9	1.5
India	133	21.4
Japan	48	7.7
Netherlands	11	1.8
Taiwan	5	0.8
United Kingdom	10	1.6
United States	344	55.5
Other countries	45	7.3

**Table 3:** Musicianship of the participants.

**Question**	**No (%)**	**Beginner (%)**	**Advanced (%)**	**Professional (%)**	**Mean (SD)**
Are you a musician?	29.0	24.2	27.9	18.9	2.37 (1.09)

The mean, SD, and median for the LS-7 and PS-4 questions are provided along with the list of questions in Appendix 1 in Supplementary Material.

Table 4 shows responses to Q9 (Enforced restriction) and Q10 (Self restriction); the perceived strictness of the guidelines of measures against COVID-19 (i.e., SiP, social distancing, and self-quarantine) enforced in the participants' region, and how strictly each participant followed the guidelines. About 85% answered that the enforced guidelines were rather strict (i.e., moderately or extremely), and they followed the guidelines rather strictly. This indicates that the participants saw the severity of pandemic, and their lifestyle was restricted accordingly.

**Table 4:** Responses on the Strictness of Guidelines for Measures against COVID-19.

**Question**	**Not at all (%)**	**A little (%)**	**Moderately (%)**	**Extremely (%)**	**Mean (SD)**
Enforced restriction	<1	15	57	28	3.1 (0.7)
Self restriction	1	13	44	42	3.3 (0.7)

Table 5 describes the responses to key LS-7 questions. These key questions were selected according to the factors found in the next section. Table 6 describes the responses to the key PS-4 questions. Most of the questions in Table 5 have normally distributed responses, with a peak at “No change” and tapering to both ends, except that Q14 “Do you stay at home more or less than before, since the COVID-19 outbreak? (Stay home)” indicated that 33.5% of participants stayed home much more.

**Table 5:** The distributions of responses to key questions employing LS-7 (seven-point Lickert scale).

**Question**	**#**	**Much less (%)**	**Less (%)**	**A little less (%)**	**No change (%)**	**A little more (%)**	**More (%)**	**Much more (%)**	**Mean (SD)**
**Life factor**									
Stay home	14	2.9	12.9	13.2	10.5	9.0	17.9	33.5	5.0 (2.2)
**Stress factor**									
Stress level change	30	4.8	12.9	17.9	23.4	19.2	14.0	7.7	4.1 (1.6)
Work risk	31	7.7	13.7	16.9	21.5	19.4	11.3	9.5	4.0 (1.7)
Annoyed with neighbors' sounds	29	10.0	16.0	18.5	36.8	9.4	6.5	2.9	3.5 (1.5)
**Music factor**									
Longer music listening	33	3.9	16.1	15.8	18.5	17.4	18.2	10.0	4.2 (1.7)
Musical activities	51	3.2	11.8	16.0	30.3	19.5	12.4	6.8	4.1 (1.5)
Attention to music	47	2.9	9.7	10.0	34.7	18.1	14.0	10.6	4.4 (1.5)
Emotional with music	46	2.6	11.9	12.9	34.0	19.0	13.5	6.0	4.2 (1.4)
Attention to environmental sounds	36	3.4	13.7	14.0	25.8	20.6	13.7	8.7	4.2 (1.6)
Moderate emotion with music	50	2.9	12.6	12.4	27.7	18.9	16.3	9.2	4.3 (1.6)
Participate in online music community	56	9.2	11.0	14.0	38.2	13.5	9.4	4.7	3.8 (1.5)

**Table 6:** Responses to key questions employing PS-4 (four-point positive-sided ordinal scale), both belonging to life factor.

**Question**	**Not at all (%)**	**A little (%)**	**Moderately (%)**	**Very much (%)**	**Mean (SD)**
Neighbors' noise level	12.3	34.7	32.9	19.7	2.60 (0.94)
Distraction from the residents	22.6	23.7	38.5	14.7	2.46 (1.00)

Table 7 summarizes the responses to Q60, “What kind of activities do you miss most?” About 35.0% of people answered that they missed outdoor festivals, followed by taking lessons (31.1%) and group rehearsals (27.7%), and so on.

**Table 7:** List of the missed musical activities and the ratio of people who answered “yes.”

**Activity**	**Ratio (%)**
Outdoor festivals	35.0
Taking lessons	31.1
Group rehearsals	27.7
Attending live performances	17.4
Performing in live performances	9.0
None	2.9

### 3.2. Factor Analysis

We selected 26 numerical variables from questions regarding the responses during the pandemic. All question phrases including either “during SiP” or “after the outbreak” were subjected to factor analysis, as shown in Table 8. All data were normalized before being entered into the factor analysis. We conducted the Kaiser-Meyer-Olkin test (0.892) and Bartlett test (*p* < 0.001) to check the adequacy of the data. Because we assume that the factors are related to each other, promax rotation was used for the analysis. The maximum likelihood estimation was used for the fitting method. We created a scree plot to determine the final number of factors. Three factors were identified and interpreted as music, lifestyle, and stress factors, where the amount of variance explained was 15%, 10%, and 9%, respectively. The major variables contributing to each factor are: ModerateEmotionWithMusic and AttentiveMusic for music factor (Q50, Q47), NeighborsNoiseLevel and StayHome for lifestyle factor (Q28, Q14), and StressLevelChange and NeighborsSoundAnnoyance for stress factor (Q30, Q21).

**Table 8:** Results of factor analysis (number of factors = 3).

**Variable name**	**Factors**		
	**Music**	**Lifestyle**	**Stress**
ModerateEmotionWithMusic	**0.72**	−0.09	0.13
MusicalActivities	**0.7**	−0.05	0.07
AttentiveMusic	**0.69**	−0.08	0.09
LongerMusicListening	**0.65**	−0.3	−0.06
EmotionalWithMusic	**0.62**	−0.13	0.21
MusicListeningPrivate	**0.52**	0.17	−0.28
AttentiveEnvSound	**0.43**	−0.18	0.3
MusicListeningWork	0.38	0.35	−0.17
ChangeTypeActivity	0.36	0.28	0.17
LikeMusic	0.34	−0.08	−0.08
OnlineMusicActivity	0.33	0.21	0.27
SelfRestriction	0.2	−0.09	0.03
NeighborsNoiseLevel	−0.03	**0.78**	0.28
DistractionResidents	−0.09	**0.67**	0.3
StayHome	0.23	**−0.51**	0.17
Musician	0	**0.49**	0.09
IncomeChange	0.2	−0.39	0.19
NoPeopleLivingWith	0.06	0.39	−0.08
NoChildren	0.01	0.36	−0.11
WorkHoursDuringSiP	0.03	−0.31	0.02
HouseHoursDuringSiP	0.07	−0.25	0.06
SelfHoursDuringSiP	0.03	−0.2	0.08
StressLevelChange	0.01	−0.04	**0.66**
NeighborsSoundAnnoyance	−0.01	0.17	**0.65**
WorkRisk	0.04	−0.02	**0.62**
WorryDuringSiP	0.31	−0.05	0.36

*Responses to the questions regarding the musical activities change, living situations, and psychological status during the SiP were submitted to the factor analysis. The major variables with the coefficients greater than 0.4 (emphasized with boldface) were submitted to the SEM analysis*.

### 3.3. Structural Equation Modeling

To conduct SEM path analysis, the three factors (music, lifestyle, and stress) found in the factor analysis were regarded as latent variables, and the responses to the questions (e.g., StressLevelChange, StayHome, ModerateEmotionWithMusic, etc.) were regarded as observed variables. We developed an initial model in which each latent variable had paths to the observed variables obtained in the factor analysis. All relationships between variables were statistically tested, and the SEM algorithm found the best-fit structure. Before conducting the SEM path analysis, all the data were normalized. The variance inflation factor among the variables in the path diagrams are calculated, and the multicollinearity across the variables was not confirmed. The coefficients of the relationships were calculated using maximum likelihood estimation. SPSS Amos 27 software was used for all analyses.

**Figure 1:**
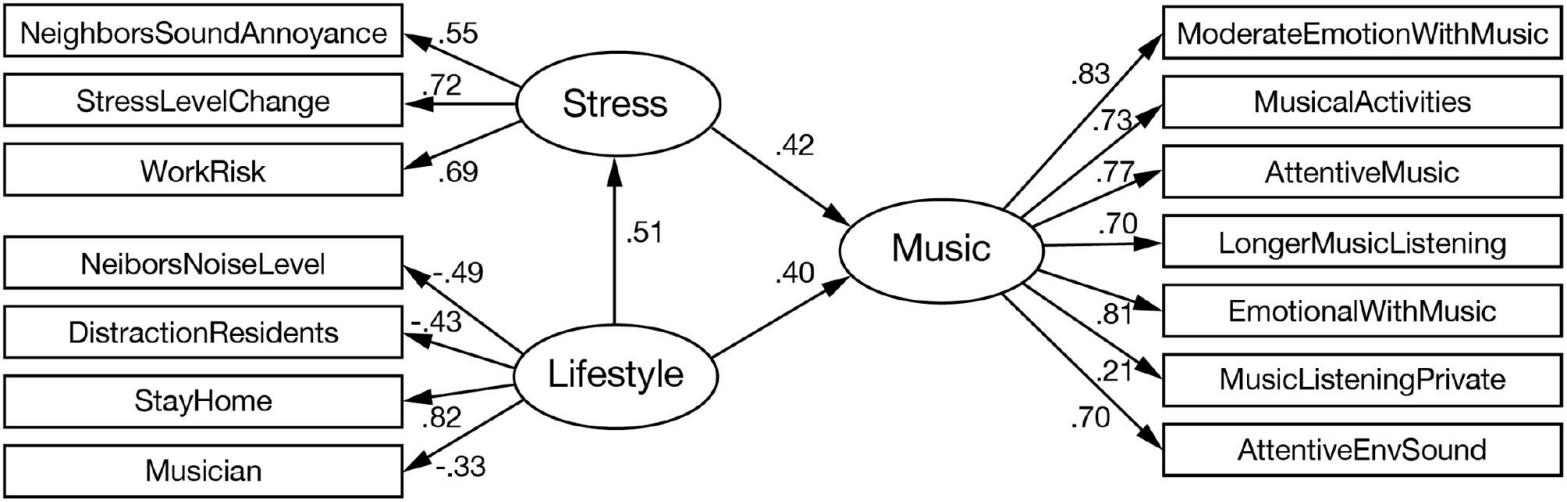
Path diagram of structural equation model. Ovals indicate latent variables, and rectangles indicate observed variables (error variables are omitted). The path between constructs indicates the direction of causality with the coefficients. A higher coefficient suggests a higher contribution to the variable.

Figure 1 shows the results of the SEM analysis. According to the model, lifestyle and stress variables significantly contribute to explaining music variable: All three paths connecting the latent variables had coefficients above 0.4 and were statistically significant (Fit indices of the model were as follows: χ^2^ = 372.870, *df* = 74, *p* < 0.001, CFI = 0.903, RMSEA = 0.081, BIC = 572.192).

The path coefficients connecting the latent variables indicate the covariance among them, and the direction of the path (arrow) indicates causality. In our model, lifestyle factors affect the stress factor, and both stress and lifestyle jointly affect the music factor. Higher coefficients for the paths between the latent and observed variables indicate the dominant variables for each factor. The model suggests that StressLevelChange, StayHome, ModerateEmotionWithMusic, and MoreEmotionalWithMusic were dominant for the stress, lifestyle, and music factors, respectively.

### 3.4. Changes in Lifestyle and Perceived Stress Level

The primary variable analyzed in this section is stress-level change (SLC, Q30). It asked whether people experienced an increased or decreased level of stress during the SiP period (PS-7, decreased- 1, no change-4, increased-7). The data were normally distributed with a mean of 4.13 (SD = 1.6), suggesting that, while many people experienced an increased level of stress, a comparable number of people experienced a similar or decreased level of stress during the SiP. This observation agrees with an earlier observation that not all individuals experienced increased stress levels (Pierce et al., 2020). Overall, gender had a significant impact on SLC (Kruskal-Wallis ANOVA, *df* = 3, χ^2^ = 12.7, *p* < 0.01). The *post-hoc* analysis indicated that females tended to express a greater level of SLC than males (Dunn's test, *p* < 0.05, Bonferroni-corrected). None of the other pairs was significantly different. In our data, age did not show a systematic influence on SLC, contrary to earlier reports that younger individuals tended to exhibit a higher level of distress (Pieh et al., 2020).

Other variables related to changes in lifestyle that were measured as continuous or quasi-continuous scales were submitted to a multiple regression model to predict SLC. The variables included in this analysis were SiPDuration (Q2), NoPeopleLivingWith (Q11), StayHome (Q14), WorkHoursBeforeSiP (Q19), WorkHoursDuringSiP (Q20), HouseHoursBeforeSiP (Q21), HouseHoursDuringSiP (Q22), SelfHoursBeforeSiP (Q23), SelfHoursDuringSiP (Q24), WorkRisk (Q31), and IncomeChange (Q32). The regression analysis comparing the full model and the best-fit model (backward removal method) accounted for 30.5% of the total variance (see Table 9). The residuals of this model were normally distributed. Among the included factors, three had a large impact. SLC tended to be higher when the risk at work (i.e., layoff, infection, and other factors) increased, the income decreased, and when people stayed at home for more hours due to the outbreak.

**Table 9:** Results of multiple regression analysis.

**Variables**	**Full model**				**Best model**			
	**B**	**SE B**	**β**	**p**	**B**	**SE B**	**β**	**p**
Intercept	1.29	0.27	.	<0.01	1.32	0.2	.	<0.0001
Work risk	0.35	0.04	0.38	<0.0001	0.35	0.04	0.38	<0.0001
Income change	0.15	0.05	0.13	<0.01	0.16	0.05	0.14	<0.001
Stay at home more or less	0.13	0.03	0.16	<0.0001	0.15	0.03	0.18	<0.0001
Working hours before SiP	−0.01	0	−0.14	<0.05	−0.01	0	−0.13	<0.01
Working hours during SiP	0.01	0	0.09	<0.05	0.01	0	0.09	<0.05
House work hours before SiP	0	0	0.01	.	.	.	.	.
House work hours during SiP	0	0	0.02	.	.	.	.	.
Self hours before SiP	0.01	0	0.06	.	0.01	0	0.38	<0.05
Self hours during SiP	0	0	0.02	.	.	.	.	.
Duration of SiP Week	0.03	0.02	0.06	.	.	.	.	.
Number of people living with	−0.01	0.04	−0.01	.	.	.	.	.
		*R* ^2^	0.31			*R* ^2^	0.30	
		*R* ^2*^	0.30			*R* ^2*^	0.30	
		F	24.58	<0.0001		F	44.66	<0.0001

Responses regarding lifestyle changes in continuous and quasi-continuous scales were submitted to a multiple regression model to predict stress-level change. The full model and the best model parameters (B is coefficient, SE B is standard errors, β is standardized coefficient, R^2*^ is adjusted R^2^).

It appeared that the perceived strictness of the regulations enforced by the local government had a significant effect on SLC as shown in Table 10 [Kruskal-Wallis ANOVA, *df* = 2, χ^2^ = 7.75, *p* < 0.05; we removed nine responses in the analyses in this paragraph that indicated no restrictions to one of the two questions: EnforcedRestriction (Q9), SelfRestriction (Q10)]. There was a trend of SLC decreasing as the enforced restrictions became stricter. In particular, *post-hoc* analyses indicated that SLC of the small-restriction group was significantly greater than that of the extremely strict group (Dunn's test, *p* < 0.05, Bonferroni-corrected). SLC response of the moderately-strict group did not differ significantly between the two groups. Interestingly, the degree of self-restriction showed a significant reverse tendency (Kruskal-Wallis ANOVA, *df* = 2, χ^2^ = 10.40, *p* < 0.01), as shown in Table 10. In general, the more strictly individuals followed the rules, the higher their SLC. In particular, *post-hoc* analyses indicated that the small-self-restriction group had significantly lower levels of SLC than the groups that were extremely self-strict (Dunn's test, *p* < 0.05, Bonferroni-corrected). The same analysis indicated that the small-self-restriction group tended to show lower levels of SLC than the moderate-self-restriction group, but the difference was marginally non-significant (*p* = 0.08 after the correction).

**Table 10:** Average stress-level change (SLC) by three levels of restrictions (enforced restriction vs. self restriction).

**Variables**	**Enforced restriction**			**Self restriction**		
	***N***	**Mean SLC**	**SD**	***N***	**Mean SLC**	**SD**
Small restriction	95	4.4	1.62	79	3.6	1.5
Moderately strict	346	4.2	1.47	272	4.1	1.47
Extremely strict	170	3.9	1.82	260	4.3	1.75

Whether people started to live with others due to the outbreak (Q13) had a significant influence on SLC (Mann-Whitney *U*-test, χ^2^ = 31.81, *p* < 0.001). People who answered yes to this question (*N* = 280, mean SLC = 3.7) indicated significantly lower SLC than those who did not (*N* = 338, mean SLC = 4.4).

Moreover, the number of children living together seems to affect this factor. There was a significant negative correlation between SLC and the number of children living together when people started living with others because of the outbreak (ρ = −0.22, *p* < 0.001), whereas such a trend was not observed when people did not change the members of their households [ρ = −0.1, *p* = 0.07(*n*.*s*.)]. This most likely indicates that people became less stressed when they started to live with their children after the outbreak. The frequency of distraction a person is subject to from the residents of one's household also showed a slight but negative correlation with SLC (ρ = −0.12, *p* < 0.05). Interestingly, this indicates that people who had more distractions from the residence tended to perceive slightly less stress.

### 3.5. Stress Level Changes and Functionality of Music

In order to analyze the functional use of music during SiP, we asked how participants used music in their daily lives before and during SiP (FunctionMusicBeforeSiP and FunctionMusicDuringSiP, Q48, and Q49, respectively) in the checklist format. The list of music functions contained the following nine items: moderate emotion, influence mood, concentrate, relax, be creative, have fun, fall asleep, play with friends, and play with children. Participants could select as many categories as they liked. A simple before-during frequency comparison using a sign test, as shown in Table 11, indicated that people tended to use music significantly more during the SiP to fall asleep and play with children. The same analysis revealed that people used music significantly more before the SiP to have fun and to play with friends.

**Table 11:** The functional use of music before and after shelter-in-place.

**Functions**	**Before SiP**	**During SiP**
Moderate emotion	241	267
Influence my mood	292	298
Concentrate	237	256
Relax	437	437
Be creative	217	221
Have fun	354	323
Fall asleep	143	173(^*^)
Play with friends	161	118^**^
Play with children	103	127(^*^)

Frequency counts for 9 music functions (*indicates significant increase or decrease of frequencies between before and during SiP by sign test, ^**^p < 0.01, (^*^)p < 0.1, Holm-Bonferroni corrected).

Next, we examined the associations between the functions of music and SLC. Responses to the nine functions of music before and during SiP (total 18 categories) were coded as 0 (no) and 1 (yes), and we computed the correlations (Spearman's ρ) between these musical functions and SLC (see Table 12). While many functions did not show significant correlations with SLC, the functions that concerned mental well-being, such as dealing with one's emotions, mood, relaxation, and fun, tended to correlate with SLC. Furthermore, using music to moderate emotion was significantly correlated with SLC during SiP, but not before SiP, suggesting that the way people use music changed during the outbreak and the frequency of using music for this purpose corresponded well with SLC.

**Table 12:** Correlation (Spearman's ρ) between stress-level change (SLC) and 9 functions of music before and during SiP ^*^p < 0.05, (^*^)*p* < 0.1, Holm-Bonferroni corrected.

**Functions**	**Before SiP**	**During SiP**
Moderate emotion	0.05	0.21^*^
Influence my mood	0.15^*^	0.17^*^
Concentrate	0.04	0.02
Relax	0.11(^*^)	0.12(^*^)
Be creative	−0.01	0.03
Have fun	0.05	0.08
Fall asleep	0.00	0.03
Play with friends	0.07	−0.01
Play with children	−0.01	−0.01

### 3.6. Stress, Housing, and Reaction to Environmental Sounds

In order to investigate the experience of noise and environmental sounds during SiP, we asked questions about housing types (as a mean to describe the acoustical isolation between houses), perceived noise level, attention to environmental sounds, and annoyance with neighbors' sounds.

Table 13 shows the relationship between the housing types (Q27) and noise level (Q28), and attention to environmental sounds (Q36). For these two questions, differences of mean scores across house type was significant [Kruskal-Wallis ANOVA, *df* = 4, χ^2^ = 99.3 (Q27), χ^2^ = 40.2 (Q28), *p* < 0.001]. For the noise level, *post-hoc* tests showed that most of the possible pairs were significantly different (Dunn's test, *p* < 0.05, Bonferroni corrected), except the following two pairs; collective housing and semi-detached house, and dormitory and studio. For the attention to environmental sounds, *post-hoc* tests showed that most of the possible pairs were significantly different (Dunn's test, *p* < 0.05, Bonferroni corrected), except the pairs of collective housing and semi-detached house, collective housing and studio, dormitory and studio, and semi-detached house and studio. These indicate that the smaller the type of housing is, people hear neighbors' noise more clearly and are less attentive to environmental sounds.

**Table 13:** Housing type and environmental sounds.

**Question**	**All**	**Detached house**	**Collective housing**	**Semi-detached house**	**Studio**	**Dormitory**
Clarity of neighbors' noise	2.60 (0.94)	2.15 (0.97)	2.62 (0.82)	2.74 (0.85)	3.20 (0.80)	3.55 (0.69)
Attention to environmental sound	4.21 (1.58)	4.67 (1.39)	4.20 (1.57)	4.00 (1.59)	3.71 (1.71)	3.08 (1.48)

*Mean scores and SD for clarity of neighbors' noise and attention to environmental sounds*.

The noise level differences due to housing type do not necessarily relate to annoyance due to neighbors' sounds (Q29), although we initially expected that. The difference in the mean annoyance score, grouped by housing type, was not significantly different (Kruskal-Wallis ANOVA, *df* = 4, χ^2^ = 7.45, *n*.*s*.), and the correlation between annoyance and noise clarity was not significant (Spearman's ρ = 0.012, *p* = 0.77). Rather, annoyance was clearly correlated with SLC (Q30) (Spearman's ρ = 0.42, *p* < 0.05), and with attention to environmental sounds (Q36; Spearman's ρ = 0.35, *p* < 0.05).

Table 14 provides the correlations between the variables on perception and recognition of noise, environmental sounds and music, i.e., neighbors' noise level (Q28), neighbors' sound annoyance (Q29), more attentive to environmental sounds (Q36), more emotional with music (Q46), and more attentive to music (Q47).

**Table 14:** Correlations between the variables on perception and recognition of noise, environmental sounds, and music.

**Variable**	**Neighbors noise level**	**Neighbors sound annoyance**	**Attentive env sound**	**Emotional with music**	**Attentive music**
Neighbors noise level	–			
Neighbors sound annoyance	0.012	–			
Attentive env sound	−0.202^***^	0.349^***^	–		
Emotional with music	−0.156^***^	0.295^***^	0.618^***^	–
Attentive Music	-0.100^*^	0.243^***^	0.504^***^	0.659^***^	–

*Numbers indicate Spearman's ρ*,

* *p < 0.05,*

*** *p < 0.001*.

Most of these variables correlate significantly, except for the Q28 and Q29 pairs. The high correlations among responses for attention to environmental sounds, attention to music, and being more emotional with music may indicate that the changes in the perception of music and environmental sounds co-occurred during SiP.

### 3.7. Multi-Group SEM Based on Musicianship

Multi-group SEM was performed to capture participants' different response patterns depending on their musicianship. In order to examine the common structure for the groups based on musicianship, the goodness of fit is calculated for all the groups applying various candidate-models. As a result, we did not find a single structure to represent all the groups: The constraints of measurement invariance and structural invariance were not confirmed, suggesting that each group has a different path structure. Afterwards, we calculated the path diagram for each group using the standard SEM sprocedures.

**Figure 2:**
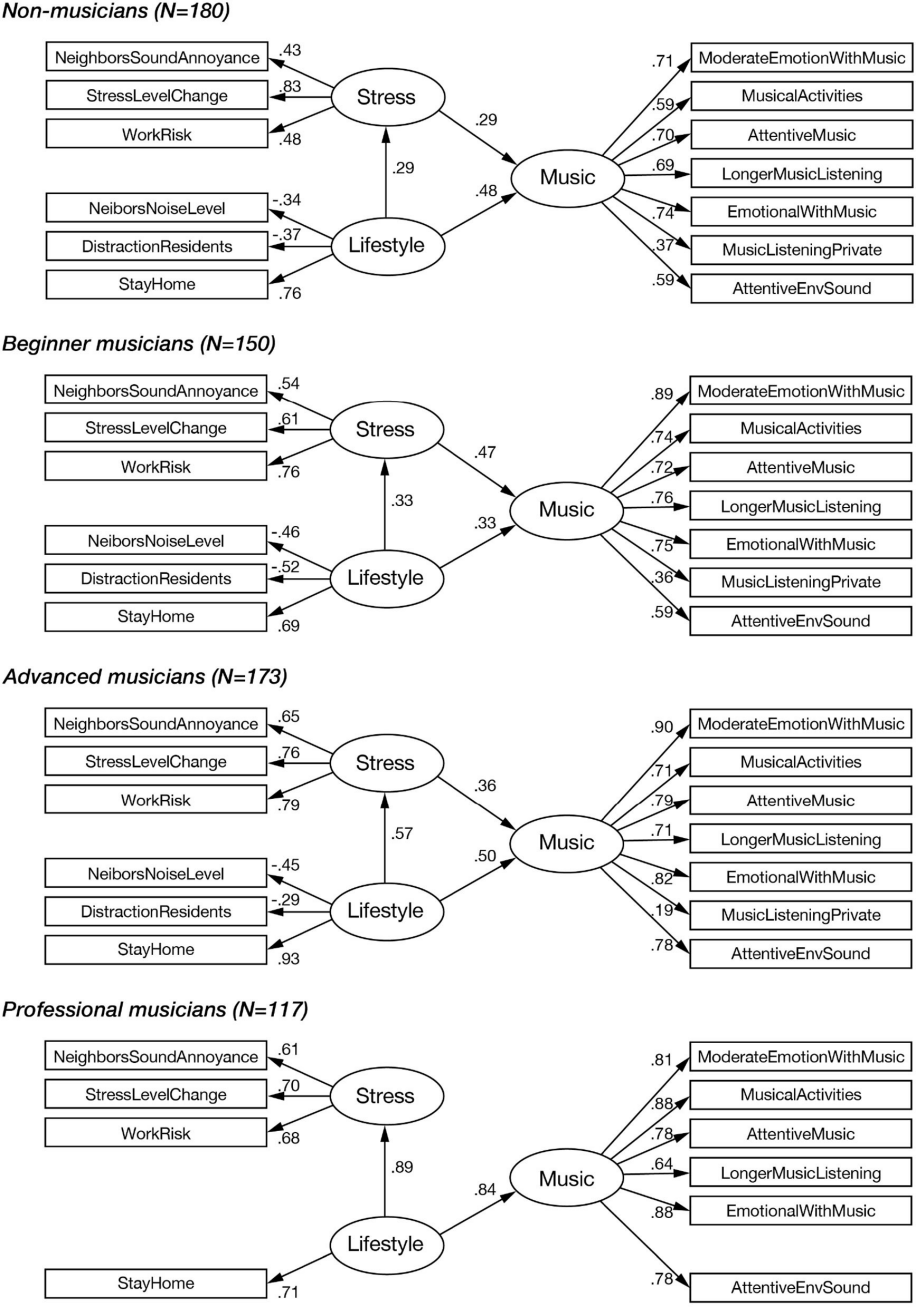
Path diagrams of Multi-group SEM among musicianship. Responses were divided on the musicianship level, and the SEM analysis was applied for each group.

Figure 2 shows the SEM path diagrams for each group, and Table 15 shows the fit indices. The structure of the professional musician group differs from that of the other groups. In the professional musician group, some paths that are present in the other groups are missing, specifically the path from stress factor to music factor, the paths from LifeStyle to NeighborNoiseLevel and DistractionResidents, and the path from music factor to MusicListeningPrivate.

**Table 15:** Fit indices of path analyses of structural models on multiple groups based on musicianship.

**Group**	***N***	**Indices**					
		**χ^2^**	**df**	**p-value**	**CFI**	**RMSEA**	**BIC**
Non-musicians	180	148.349	62	<0.001	0.847	0.088	298.945
Beginner musicians	150	122.604	62	<0.001	0.908	0.081	267.912
Advanced musicians	173	161.184	62	<0.001	0.905	0.096	310.629
Professional musicians	117	60.671	33	<0.001	0.956	0.085	165.438

This structure implies that the StayHome variable was the only component that caused all the changes in stress and music among professional musicians. Besides StayHome, other variables related to the living environment, such as DistractionResidents and NeighborNoiseLevel, disappear from the life factor for this group, suggesting that these variables do not affect their musical behaviors. In addition, the stress factor did not cause a change in musical behaviors (i.e., the arrow from stress to music is missing). All these findings suggest that professional musicians were dominantly impacted by the StayHome aspect.

### 3.8. Musicianship and Musical-Emotion Related Responses

The multi-group SEM analysis based on musicianship showed the musical-emotion related variables such as ModerateEmotionWithMusic and EmotionalWithMusic had higher coefficients. However, multi-group SEMs cannot be directly compared across groups. This section provides details of the musical-emotion-related questions based on musicianship.

Table 16 shows the mean scores and SD of responses to the musical-emotion related questions, where NM refers to non-musicians. For the question “Music felt more emotional during SiP (Q46),” the mean score decreases with more-experienced musicianship, and increases with the professional musician group. The difference of mean scores among groups is significant (Kruskal-Wallis ANOVA, *df* = 3, χ^2^ = 16.3, *p* < 0.001). The *post-hoc* analysis (Dunn's test with Bonferroni correction) showed that the differences between the pairs of Non-musician and Advanced, Non-musician and Professional, and Beginner and Advanced are significant (*p* < 0.05), while the other pairs were not significantly different.

**Table 16:** Mean and SD of responses to musical-emotion related questions, grouped by musicianship.

**Question**	**All**	**NM**	**Beginner**	**Advanced**	**Professional**
Emotional with music (Q46)	4.20 (1.44)	4.47 (1.17)	4.32 (1.46)	3.91 (1.48)	4.02 (1.63)
Moderate emotion with music (Q50)	4.33 (1.57)	4.61 (1.39)	4.53 (1.58)	3.91 (1.49)	4.24 (1.77)

The same pattern was observed for the “Moderate emotion with music (Q50),” and the difference in mean scores among groups was significant (Kurskal-Wallis ANOVA, *df* = 3, χ^2^ = 21.3, *p* < 0.001). The *post-hoc* analysis (Dunn's test with Bonferroni correction) showed that the mean score of Non-musician was significantly higher than that of Advanced, and the mean score of Beginner was significantly higher than that of Advanced (*p* < 0.001), while the other pairs were not significantly different.

### 3.9. Musicianship, Music Listening Time, and Media Usages

Table 17 shows the cross tabulation of music listening frequency before SiP, during SiP (Q34, Q35, respectively), and the difference between them. While NM and Beginner increased the percentage of people who listened to music frequently, advanced and professional musicians did not show much difference.

**Table 17:** Distribution of music listening frequency before and during SiP (shelter-in-place) grouped by musicianship.

**Musicianship**	**Less than once a month (%)**	**Twice a month (%)**	**1–2 times a week (%)**	**3–5 times a week (%)**	**Less than 1 h, daily (%)**	**1–3 h daily (%)**	**More than 3 h daily (%)**
**Frequency of music listening before SiP**							
Non-musician	0.6	1.1	8.3	17.2	31.1	32.2	9.4
Beginner	0.7	0.0	5.3	20.7	26.7	35.3	11.3
Advanced	0.0	0.0	4.0	22.5	20.8	37.6	15.0
Professional	0.9	0.9	4.3	12.0	15.4	38.5	28.2
**Music listening during SiP**							
Non-musician	1.7	1.1	7.2	6.1	16.7	38.9	28.3
Beginner	1.3	0.0	4.0	13.3	11.3	36.7	33.3
Advanced	0.0	0.6	6.9	16.8	20.2	37.0	18.5
Professional	0.9	0.9	9.4	10.3	13.7	37.6	27.4
**Difference**							
Non-musician	1.1	0.0	−1.1	−11.1	−14.4	6.7	18.9
Beginner	0.7	0.0	−1.3	−7.3	−15.3	1.3	22.0
Advanced	0.0	0.6	2.9	−5.8	−0.6	−0.6	3.5
Professional	0.0	0.0	5.1	−1.7	−1.7	−0.9	−0.9

Table 18 shows the mean scores for the questions about music listening time change grouped by musicianship. The mean scores of longer music listening time (Q33) were different depnding on musicianship (Kruskal-Wallis ANOVA, *df* = 3, χ^2^ = 26.7, *p* < 0.001). The *post-hoc* analysis (Dunn's test with Bonferroni correction) showed that the differences were significant between Non-musician and Advanced (*p* < 0.001), Non-musician and Professionals (*p* < 0.001), and Beginner and Advanced (*p* < 0.005), while the other pairs were not significantly different.

**Table 18:** Mean and SD of responses to music listening- and activity-related questions, grouped by musicianship.

**Question**	**All**	**NM**	**Beginner**	**Advanced**	**Professional**
Music listening time	4.24 (1.70)	4.69 (1.64)	4.38 (1.75)	3.86 (1.56)	3.93 (1.78)
Music via YouTube	4.45 (1.62)	4.92 (1.40)	4.53 (1.65)	4.14 (1.58)	4.05 (1.76)
Music via streaming	4.35 (1.56)	4.76 (1.40)	4.48 (1.52)	3.98 (1.53)	4.11 (1.69)
Music via TV	4.08 (1.43)	4.38 (1.28)	4.09 (1.56)	3.90 (1.25)	3.89 (1.67)
Music via radio	3.96 (1.50)	4.16 (1.51)	3.97 (1.37)	3.78 (1.38)	3.89 (1.76)
Musical activity	4.15 (1.48)	4.40 (1.23)	4.21 (1.57)	3.94 (1.49)	4.03 (1.67)
Online music activity	3.83 (1.52)	3.63 (1.31)	3.72 (1.49)	3.88 (1.47)	4.18 (1.84)

Observing each medium, mean scores for longer music listening via YouTube (Q40) and streaming (Q37) increased, followed by TV (Q39), while radio listening (Q38) decreased during SiP.

The mean scores of longer YouTube watch time (Q40) were different depending on musicianship (Kruskal-Wallis ANOVA, *df* = 3, χ^2^ = 27.5, *p* < 0.001), with *post-hoc* analysis (Dunn's test with Bonferroni correction) showing significant differences between Non-musican and Advanced (*p* < 0.001), and Non-musician and Professional (*p* < 0.001), while the other pairs were not significantly different.

The mean scores of longer streaming time (Q37) were different depending on musicianship (Kruskal-Wallis ANOVA, *df* = 3, χ^2^ = 26.3, *p* < 0.001), with *post-hoc* analysis (Dunn's test with Bonferroni correction) showing significant differences between Non-musician and Advanced (*p* < 0.001), Non-musician and Professional (*p* < 0.001), and Beginner and Advanced (*p* < 0.05), while the other pairs were not significantly different.

The mean scores of longer TV time (Q39) were different depending on musicianship (Kruskal-Wallis ANOVA, *df* = 3, χ^2^ = 13.0, *p* < 0.005), with *post-hoc* analysis (Dunn's test with Bonferroni correction) showing significant differences between Non-musician and Advanced (*p* < 0.01), and Non-musican and Professional (*p* < 0.05), while the other pairs were not significantly different.

The mean scores of longer Radio time (Q38) were not different depending on musicianship (Kruskal-Wallis ANOVA, *df* = 3, χ^2^ = 5.6, *n*.*s*.).

Although the media usage analysis suggested increased use of Internet media, participation in the online musical community (Q56) had an opposite pattern to the above, even compared with musical activity (Q51), following a pattern similar to that of music listening. The mean scores for participation in the online musical community were significantly different depending on the musicianship (Kruskal-Wallis ANOVA, *df* = 3, χ^2^ = 8.25, *p* < 0.05), with *post-hoc* analysis (Dunn's test with Bonferroni correction) showing significant differences between Non-musican and Professional (*p* < 0.05), and Beginner and Professional (*p* < 0.05) while the other pairs were not significantly different. The mean scores for musical activities were significantly different depending on the musicianship (Kruskal-Wallis ANOVA, *df* = 3, χ^2^ = 11.73, *p* < 0.01), with *post-hoc* analysis (Dunn's test with Bonferroni correction) showing significant differences between Non-musican and Advanced (*p* < 0.01), and Non-musician and Professional (*p* < 0.05), while the other pairs were not significantly different.

In summary, less-experienced musicians tended to increase music listening time on YouTube and streaming media during SiP, while more-experienced musicians tended to participate more in online music communities with interaction, suggesting that experienced musicians may have preferred more proactive musical behaviors, rather than passively consuming musical content. The way people use the Internet for musical purposes seems to differ depending on musicianship.

## 4. Discussion

In June 2020, we conducted an online survey to investigate the association between lifestyle, psychological status, and musical behaviors during the SiP period of the COVID-19 pandemic's first wave. The responses of 620 participants from 24 countries were analyzed. The responses were mainly from the U.S. (55.5%) and India (21.4%), followed by other countries such as Japan (7.7%), the Netherlands (1.8%), the United Kingdom (1.6%), and so on. Based on factor analysis, our SEM approach with a triangular structure revealed that two of the three latent variables, namely, stress (stress-level change, work risk, feeling annoyed with neighbors' noise) and lifestyle (increase in stay home time, co-residents, and living environment), directly and indirectly influenced the third variable, musical behaviors (moderating emotion with music, feeling emotional with music, being more attentive to music, having more musical activities, etc.). The causal connections drawn in our model are useful for explaining many formal and informal observations, such as the effect of individuals' employment situation on music listening during SiP (Cabedo-Mas et al., 2021). Interestingly, one's musicianship seems to influence the balance among the three latent variables.

The questions covered a wide range of information to capture the unique moment, and consequently the format of the questions was not always uniform, making it sometimes hard to compare. Some of the data were already retrospective at the time of survey, resulting in potentially unreliable judgments. Furthermore, the stress, lifestyle, and music factors explained the variables for 15, 10, and 9% respectively, implying the presence of some other factors which were not detected in this survey. Nevertheless, we will focus on significant variables and observations in detail to give a better overview of our results.

### 4.1. Stress

This section focuses on the findings related to the stress latent variable of the model. All SEM results indicate that there is a causal connection from lifestyle to stress latent variables. Among the variables that constitute stress, SLC is the most critical. The detailed analysis of SLC highlighted that fundamental factors for one's everyday life, such as work risk and income change, are critical for SLC, in agreement with previous findings (Pieh et al., 2020). In addition to these fundamental factors, we identified two types of social variables that are correlated with SLC. One type is related to regulation, and the other is related to contact with people. People living in areas with stricter regulations had lower SLC. We think that more stringent regulations mean tighter control of the situation, and during the COVID-19 pandemic, people feel more secure in these areas. The other type is contact with people. Three variables related to this type showed significant correlations with SLC (Q12-NoChildren, Q13-LiveWithOthersDueToOutbreak, Q25-DisctractionResidents), indicating that the greater the number of contacts, the lower their SLC. This agrees with the alarm published in May 2020 that social isolation, connection, and intimate relationships would have a significant influence on stress (Bavel et al., 2020).

In general, there is a significant causal connection from the stress to the music latent variable, suggesting that an increase in perceived stress causes more frequent musical activities and engagements in music. Interestingly, this connection was missing in professional musicians (see section 4.3).

People use music in different ways, such as to relax, have fun, influence mood, moderate emotion, concentrate, and so on. We found that the pandemic shifted the frequency of music usage (Table 11). In particular, there was a significant decrease in “play with friends” and marginal increases in “fall asleep” and “play with children.” The decrease in “play with friends” most likely reflects the loss of social opportunity outside the home during the SiP. The increase in “play with children” seems to reflect that the adults had to spend a long time taking care of children during school closures. Researchers have reported that parents increasingly use music to moderate and enhance children's emotions during SiP (Cho and Ilari, 2021; Ribeiro et al., 2021a; Steinberg et al., 2021). We presume that our results reflect the behavior of caregivers. A somewhat surprising finding was the increased usage of music as a sleep support. This may be related to the sleep difficulties experienced by many people during the pandemic. Multiple large-scale studies reported a decrease of sleep quality during SiP (Fu et al., 2020; Pinto et al., 2020; Robillard et al., 2021). The data may suggest that more people are searching for better sleep, as listening to music is known to improve sleep quality (De Niet et al., 2009).

The data revealed that “influencing mood” is significantly correlated with SLC before and during SiP. Interestingly, “moderate emotion” and SLC were significantly correlated, but only during SiP. Although we cannot offer a concrete explanation for the difference before and during SiP, the correlations between SLC and mood and between SLC and emotion variables during SiP are in line with previous findings that stress factors predict how much music is used to regulate and influence mood (Chamorro-Premuzic et al., 2012; Getz et al., 2014; Vella and Mills, 2017). In addition, a stronger correlation between SLC and “moderate emotion” during SiP than before SiP suggests a general increase in the need for music as a support tool to deal with the pandemic-induced stress. This agrees with the recently reported positive role of music listening for emotion regulation and venting negative emotion during the pandemic (Herrero et al., 2020; Carlson et al., 2021; Ferreri et al., 2021; Granot et al., 2021; Krause et al., 2021; Mak et al., 2021; Martín et al., 2021; Martínez-Castilla et al., 2021; Ribeiro et al., 2021b; Vidas et al., 2021; Ziv and Hollander-Shabtai, 2021).

### 4.2. Lifestyle

The lifestyle variables identified by our SEM analysis are stay home, neighbors' noise level, distraction from residents, and musicianship. The SEM analyses confirmed that these variables not only influenced musical behavior but also affected stress variables. We also asked a wider range of questions regarding lifestyle. In this section, we discuss noteworthy observations.

Regarding the environmental sounds and soundscape during SiP, Bartalucci et al. (2021) and Alsina-Pagès et al. (2021) reported that listeners heard nature sounds more often during SiP, and less traffic, overflights, and mechanical/electrical sounds. Furthermore, Derryberry et al. (2020) reported that songbirds sang more actively during SiP, to occupy the frequency regions emptied by the decrease in traffic noise. These studies suggest that there were significant changes in soundscapes' acoustics, which may explain the increased attention paid to environmental sounds in our data. We also found that people who live in smaller houses hear neighbors' sounds more clearly and are less attentive to environmental sounds, but not necessarily annoyed with the noises. Contrary to our initial hypothesis, more exposure to noise did not lead to a negative emotional response. However, this observation seems to be common; for example, Bartalucci et al. (2021) reported that annoyance over various noises such as road and rail traffic, overflights, and neighborhood sounds decreased during SiP, and argued that this reduction in annoyance may be due to the decrease in traffic during the lockdown.

Interestingly, we also found a significant correlation between SLC and annoyance over neighbors' sounds, while the correlation between neighbors' noise level and annoyance was not significant. This suggests that people who are more stressed tend to be more annoyed by neighbors' noises. This is in accordance with studies on environmental noise annoyance, showing that the annoyance over environmental noise is related to mental health, noise sensitivity, and concerns about the negative effects of noise, rather than mere exposure to noise or the levels of noise (Kroesen et al., 2010; Schreckenberg et al., 2010; Hammersen et al., 2016).

Attention to environmental sounds, annoyance over neighbors' noise, attention to music, and being emotional with music seem to co-occur as shown in Table 14. One possible explanation here is that people with musical training pay the same degree of attention to noise, as noise is already incorporated into musical culture as something worth careful listening (Chion, 1983), and musicians can better understand environmental sounds (Lemaitre et al., 2010). More focused listening to both music and environmental sounds could have caused the mixed emotional responses to them. Another possibility is that the novel soundscape caused by lockdown drew attention from listeners with auditory sensitivity, while they were attentive to music to moderate emotion during SiP. It is possible that there was no shared mechanism between the perceptions of musical and environmental sounds. It is difficult to distinguish between these two in our survey, and this remains a future problem.

### 4.3. Musicianship

Musicianship was one of the important lifestyle variables in our study. We presumed that individuals' degree of musicianship would influence their psychological responses and musical behaviors during SiP, and our multi-group SEM analysis (Figure 2) highlighted some interesting differences among people with different levels of musicianship. The basic triangular structure of the latent variables (i.e., stress, lifestyle, and music) and connections are shared across different types of musicianship, except for professional musicians. Professional musicians have some missing elements in the model, compared to the other groups: first, the path from stress to music is missing, and second, the observed variables of neighbors' noise level, distraction from residents, and music listening private are also missing. This most likely suggests that professional musicians are more autonomous about their musical behaviors (i.e., stress does not affect their musical behaviors) than less-experienced musicians, and for professional musicians, music listening in private was not affected by SiP, unlike the less-experienced musicians. For example, non-musicians and beginner musicians increased the frequency of music listening during SiP, while advanced and professional musicians did not (see Table 17). Perhaps many of the professional and advanced musicians had already reached the maximum frequency for music listening before SiP, or had already established a desirable routine before SiP.

While multi-group SEM showed the lacking path from stress to music for professional musicians, they were similarly active in musical behaviors, including the use of music to moderate emotion. For example, the mean scores of “moderate emotion during SiP more often than before” by professional musicians (Table 16) was not significantly different from the other groups. This suggests the possibility that professionals approached music during SiP, not directly for stress, but for other reasons and motivations. Martínez-Castilla et al. (2021) might provide a good hint: higher musical training is related to the higher perceived importance of music, and eventually the perceived efficacy of music (e.g., venting emotion.) This example implies that professionals approach music rather rationally, based on trust and knowledge in music rather than as a direct emotional response to stress. The knowledge and experience that advanced and professional musicians possess may modify the way they perceive and use music. Such considerations remain a problem for future research.

The analysis of media usages by musicianship showed a clear contrast depending on its degree. While less-experienced musicians increased the music listening time, especially via YouTube and streaming services, more-experienced musicians increased their participation in online musical communities (Table 18). This supports our presumption that less-experienced musicians used the Internet to consume musical content, while more-experienced musicians used the Internet to produce musical content. Notably, increased YouTube and streaming service usage has also been reported (Hurwitz and Krumhansl, 2021; Krause et al., 2021). Also, musicians' struggle to shift to online music performance and production has been reported by many (Cohen and Ginsborg, 2021; Daffern and Brereton, 2021; Fram et al., 2021; MacDonald et al., 2021; Spiro et al., 2021). Hansen et al. (2021) created a database for such musical works available online. These studies show that music was a source of peace for non-musicians and beginners, but continuing music was a sometimes painful, yet innovative, struggle for advanced and professional musicians. Our analysis depicts this contrast, along with these studies.

## 5. Conclusion

Based on large-scale survey data, the current study extracted and evaluated a model to explain the impact of the COVID-19 pandemic on people's musical behavior. The SEM analysis, with a triangular model with stress, lifestyle, and music behavior as the main components, highlighted that social situations and lifestyle (including musical training) affected musical behavior changes during SiP. Our findings align with recent reports suggesting that people are coping with the difficulty of pandemics by using music in various ways, notably by taking advantage of Internet technologies.

Importantly, the survey was conducted in early June 2020, at the later stage of the first-wave lockdown. At the time of the survey, we asked a wider range of questions to capture this historical moment. Our analyses, especially the factor analysis and SEM analyses, revealed significant variables among these. It would be highly informative to conduct a follow-up study that focuses on these variables to investigate the later period of the pandemic and further validate and improve the model.

## Data Availability Statement

The datasets presented in this article are not openly available. For any inquiry about the data, please contact the correponding author.

## Ethics Statement

The studies involving human participants were reviewed and approved by the Ethics Committee of the Faculty of Library, Information and Media Science, University of Tsukuba (No. 20-6). The participants provided their written informed consent to participate in this study.

## Author Contributions

All authors contributed equally on the research design, data collection, data analysis, and preparation of the manuscript.

## Funding

This research was supported by JST Mirai Program (grant number JPMJMI19G8) and the Faculty of Library, Information and Media Science, University of Tsukuba, Japan.

## Conflict of Interest

The authors declare that the research was conducted in the absence of any commercial or financial relationships that could be construed as a potential conflict of interest.

## Publisher's Note

All claims expressed in this article are solely those of the authors and do not necessarily represent those of their affiliated organizations, or those of the publisher, the editors and the reviewers. Any product that may be evaluated in this article, or claim that may be made by its manufacturer, is not guaranteed or endorsed by the publisher.

## Footnotes

^1^ http://www.auditory.org/

^2^ https://www.facebook.com/smcnetorg

^3^ https://icad.org/mailing-list/

^4^ https://staff.aist.go.jp/h.kojima/onsei-mail.html

^5^ https://asj-eacom.acoustics.jp/mailinglist/

^6^ https://jssa.info/

^7^ http://jsmpc.org/

^8^The invitation message: “We are studying people's lifestyle and musical activities during measures against COVID-19, such as shelter-in-place, lockdown, social distance and quarantine. We would like to invite you to our ‘Music in Quarantine’ questionnaire. It will take about 20 min to complete.”
