# Supplementary material for: Music in Quarantine: Connections Between Changes in Lifestyle, Psychological States, and Musical Behaviors During COVID-19 Pandemic
Source: Front Psychol. 2021 Oct 11;12:689505. doi: 10.3389/fpsyg.2021.689505 (PMC8542664; doi:10.3389/fpsyg.2021.689505)
Supplement: Supplementary file 1 [file Data_Sheet_1.pdf]

Appendix 1. Full list of the questionnaires

A1.1. Terms used for the questionnaire

We used the following terms in the questions or the descriptions of the questionnaire.

- SiP: shelter-in-place, indicating any restriction measures that your region is taking against COVID-19 (e.g., shelter-in-place, lockdown, social distance, quarantine).
- SiP peak time: the period of strictest restrictions.
- X-point Likert scale (e.g., staying home more or less is, 1 "much less" to 7 "much more," where 4 indicates "no change"). Note that the Likert scale has positive and negative polarities.
- PS-X: X-point positive-sided ordinal scale (e.g., strictness of SiP is; no restriction, a little restriction, moderately strict, extremely strict. Note that there is no negative polarity beyond "no restriction.")

A1.2. List of questions

The following is a list of questions (question numbers and sentences) asked in the questionnaire, along with the format of the answers, and associated variable names.

1. When was the onset of the SiP peak time at your place? (Pull-down choice; SiPOnset)
2. What is the duration of the SiP peak time at your place? (Pull-down choice; SiPDuration)
3. How much were you worried about COVID-19 during the SiP peak time? (LS-5; WorryDuringSiP; mean=3.27, SD=1.30, median=3)
4. How much are your worried about COVID-19 now? (LS-5; WorryNow; mean=2.90, SD=1.24, median=3)
5. Please select the country where you live now. (Pull-down choice; Country)
6. Please enter your postal code. (Enter number; PostalCode)
7. Please select your age group. (Pull-down choice; Age)
8. Your gender (Select from list; Gender)
9. How strict are the guidelines of the measures against COVID-19 in your region (i.e., shelter-in-place, social distance, lockdown, quarantine)? (PS-4; EnforcedRestriction; mean=3.11, SD=0.66, median=3)
10. How do you follow the suggested guidelines? (PS-4; SelfRestriction; mean=3.26, SD=0.74, median=3)
11. How many people are you currently living with, including yourself? If you live in multiple locations, give an average number of people. (Select a number; NoPeopleLivingWith)

12. How many children (age under 12) are you living with? (Select a number; NoChildren)
13. Did you start living with somebody (i.e., family, partner, friends, etc.) after the COVID-19 outbreak? For example, your children came home from college, or you started living with your parents. (Yes-No; LiveWithOthersDueToOutbreak)
14. Do you stay at home more or less than before, since the COVID-19 outbreak? (LS-7; StayHome; mean=4.98, SD=1.95, median=6)
15. Are you a musician (e.g., playing musical instruments, singing, composing, DJ, audio-engineering, theorist, etc.)? (PS-4; Musician; mean=2.36, SD=1.09, median=2)
16. Do you like music? (PS-4; LikeMusic; mean=3.74, SD=0.51, median=4)
17. What are your sound playback environments? Check all that apply. (Checklist; SoundPlayback)
18. What sources do you use when you listen to music? Click all that apply. (Checklist; SourceOfMusic)
19. Before the outbreak, how many hours a week did you spend for work? Please enter a number. (Enter a number; WorkHoursBeforeSiP; mean=31.93, SD=17.63, median=40)
20. During the SiP peak time (i.e., the strictest time of measures against COVID-19), how many hours a week did you spend for work? Please enter a number. (Enter a number; WorkHoursDuringSiP; mean=25.79, SD=18.81, median=25)
21. Before the outbreak, how many hours a week did you spend for home and family related tasks and activities? Please enter a number. (Enter a number; HouseHoursBeforeSiP; mean=16.952, SD=16.60, median=10)
22. During the SiP peak time, how many hours a week did you spend for home and family related tasks and activities? Please enter a number. (Enter a number; HouseHoursDuringSiP; mean=22.87, SD=25.00, median=15)
23. Before the outbreak, how many hours a week did you spend for your leisure / relaxation / hobby / sports activities? Please enter the total number of hours. (Enter a number; SelfHoursBeforeSiP; mean=11.69, SD=10.27, median=10)
24. During the SiP peak time, how many hours a week did you spend for leisure / relaxation / hobby / sports? Please enter the total number of hours. (Enter a number; SelfHoursDuringSiP; mean=14.65, SD=14.30, median=10)
25. Do you get distracted by the residents of your household? (PS-4; DistractionResidents; mean=2.44, SD=1.01, median=3)
26. What kind of environment do you live in? (Select from list; LivingArea)
27. What kind of residence do you live in? (Select from list; AccomodationType)
28. How clearly do you hear your neighbors' sounds in your residence? (PS-4; NeighborsNoiseLevel; mean=2.59, SD=0.95, median=3)
29. Are you more annoyed with the environmental sounds from neighbors (noise, footsteps, voices, pets, etc.) than before the COVID 19 outbreak? (LS-7; NeighborsSoundAnnoyance; mean=3.50, SD=1.45, median=4)

30. How did your level of stress change after the outbreak? (LS-7; StressLevelChange; mean=4.12, SD=1.60, median=4)
31. Do you feel more risk with your work, such as layoffs, infections, and other factors, due to the outbreak? (LS-7; WorkRisk; mean=4.029, SD=1.71, median=4)
32. How did your income change after the outbreak? (LS-7; IncomeChange; mean=3.97, SD=1.39, median=4)
33. During the SiP peak time (strictest time of measures against COVID-19), did (do) you spend longer time listening to music than before the outbreak? (LS-7; LongerMusicListning; mean=4.24, SD=1.70, median=4)
34. How often did you listen to music before the outbreak? (PS-7; MusicListeningBeforeSiP; mean=5.32, SD=1.18, median=6)
35. How often did (do) you listen to music during the SiP peak time? (PS-7; MusicListeningDuringSiP; mean=5.60, SD=1.30, median=6)
36. Did (do) you become more attentive to environmental sounds (e.g., traffic sounds, birds, noise from neighbors, noise in your residence, etc.) during SiP than before? (LS-7; AttentiveEnvSound; mean=4.23, SD=1.58, median=4)
37. Did (do) you spend longer hours on music streaming services during the SiP peak time than before? (LS-7; RelativeStreamingTime; mean=4.35, SD=1.56, median=4)
38. Did (do) you listen to music on the radio more than before (both frequency and duration) during the SiP peak time? (LS-7; RelativeRadioTime; mean=3.96, SD=1.50, median=4)
39. Did (do) you watch musical contents on TV more than before (both frequency and duration) during the SiP peak time? (LS-7; RelativeTVTime; mean=4.08, SD=1.43, median=4)
40. During the SiP peak time, did (do) you watch musical contents on YouTube and other video-streaming services more than before (both frequency and duration)? (LS-7 ; RelativeYoutubeTime; mean=4.45, SD=1.61, median=4)
41. During the SiP peak time, did (do) you play music-related video games more than before? (LS-7; RelativeGameTime; mean=3.94, SD=1.41, median=4)
42. Did (do) you listen to music (through any media) while you work from home during the SiP peak time? (PS-4; MusicListeningWork; mean=3.00, SD=0.92, median=3)
43. Did (do) you listen to music in your private time during the SiP peak time? (PS-4; MusicListeningPrivate; mean=3.16, SD=0.73, median=3)
44. Did (do) you seek new musical pieces, or do you stick to the music you already knew before the outbreak? (Multiple choice; MusicTypeBeforeSiP)
45. Did (do) you seek new musical pieces, or do you stick to the music you already knew during the SiP peak time? (Multiple choice; MusicTypeDuringSiP)
46. Did (do) you feel more emotional when you listen to music during the SiP peak time? (LS-7; EmotionalWithMusic; mean=4.20, SD=1.44, median=4)

47. Did (do) you pay more attention when you listen to music during the SiP peak time? (LS-7; AttentiveMusic; mean=4.4, SD=1.51, median=4)
48. Before the outbreak, I used music to... Select all that apply. (Checklist; FunctionMusicBeforeSiP)
49. During the SiP peak time, I used music to... Select all that apply. (Checklist; FunctionMusicDuringSiP)
50. During the SiP peak time, did (do) you use music to moderate emotion or change your mood more often than before? (LS-7; ModerateEmotionWithMusic; mean=4.33, SD=1.57, median=4)
51. How much did (do) you play, sing, dance, play music-performing games (e.g., music focused video games) during SiP? (LS-7; MusicalActivities; mean=4.16, SD=1.48, median=4)
52. During SiP, when you perform music, did (do) you conduct the above performances by yourself, or with the residents of your household, or online? Check all that applies. (Checklist; MusicalInteraction)
53. Is there anything music related that you started during SiP? (Checklist; MusicalActivityStarted)
54. Is there any musical activity that you stopped during SiP? (Checkilist; MusicalActivityStopped)
55. During SiP, have your musical activities become more social (i.e., local or online community based) or more individual? (LS-7; ChangeTypeActivity; mean=3.27, SD=1.54, median=3)
56. During SiP, do you participate in online communities (private or public) where people create / perform / listen to music together with interaction more actively? (LS-7; OnlineMusicActivity; mean=3.83, SD=1.52, median=4)
57. Please describe the online creative community that you are involved in (i.e., name, platform, frequency, type of activity, URL, etc.). Please name as many as you can. You can also leave this empty if this does not apply to you. (Free description; OnlineCreativeCommunity)
58. Have you heard of any new online or neighborhood communities for music that emerged due to COVID-19? If yes, please tell us about it (i.e., name, platform, type of activity, URL, etc.). Are there any that have expanded? (Free description; COVIDOnlineCommunity)
59. Have you joined any new online or neighborhood communities for music that emerged due to COVID-19? If yes, please tell us about it (i.e., name, platform, type of activity, URL, etc.). (Free description; JoinedCOVIDOnlineCommunity)
60. What kind of musical activities do you miss most? Check all that apply. (Checklist; MissedActivity)
61. Please describe the sounds you missed most, during SiP. (Free description; MissedSounds)
62. Have you started, or have you been taking online lessons on musical instruments or singing in SiP? If so, what format? (Checklist; OnlineLessons)
63. If you are taking online lessons, do you think your skill is improving sufficiently? (Checklist; ImprovementOnlineCourse)
